# Clinicopathological and prognostic significance of PD-L1 expression in colorectal cancer: a systematic review and meta-analysis

**DOI:** 10.1186/s12957-018-1544-x

**Published:** 2019-01-04

**Authors:** Zefeng Shen, Lihu Gu, Danyi Mao, Manman Chen, Rongjia Jin

**Affiliations:** 1 The Second Clinical Medical College, Zhejiang Chinese Medical University, Hangzhou, Zhejiang China; 2 Department of General Surgery, Ningbo No. 2 Hospital, Ningbo, Zhejiang China; 3 Basic Medical College, Zhejiang Chinese Medical University, Hangzhou, Zhejiang China; 4 Affiliated Hospital of Medical School Ningbo University and Ningbo City Third Hospital, Ningbo, Zhejiang China

**Keywords:** Colorectal cancer, PD-L1 expression, Prognosis, Clinicopathological features, Meta-analysis

## Abstract

**Objective:**

To analyze the prognostic value of programmed death factor ligand 1 (PD-L1) in colorectal cancer.

**Methods:**

Electronic databases, such as PubMed, Web of Science, Embase, and Cochrane library, were searched to identify studies evaluating the PD-L1 expression and overall survival (OS) in these patients. Afterwards, the relevant data were extracted to perform the meta-analysis.

**Results:**

A total of 3481 patients were included in 10 studies. The combined hazard ratio (HR) was 1.22 (95%CI = 1.01–1.48, *P* = 0.04), indicating that high expression of PD-L1 was significantly correlated with poor prognosis of colorectal cancer. Apropos of clinicopathological features, the merged odds ratio (OR) exhibited that highly expressed PD-L1 was firmly related to lymphatic invasion (OR = 3.49, 95%CI = 1.54–7.90, *P* = 0.003) and advanced stage (OR = 1.77, 95%CI = 1.41–2.23, *P* < 0.00001), but not correlative with patients’ gender, microsatellite instability, or tumor location.

**Conclusion:**

The expression of PD-L1 can be utilized as an independent factor in judging the prognosis of colorectal cancer, and patients with advanced cancer or lymphatic invasion are more likely to express PD-L1. This conclusion may lay a theoretical foundation for the application of PD-1/PD-L1 immunoassay point inhibitors but still needs verifying by sizeable well-designed cohort studies.

**Electronic supplementary material:**

The online version of this article (10.1186/s12957-018-1544-x) contains supplementary material, which is available to authorized users.

## Introduction

Among the most common cancers worldwide, colorectal cancer ranks third, accounting for 10% of all tumor cases [1]. In 2012, the disease engendered 1,400,000 new cases and nearly 700,000 deaths [2]. According to relevant research, 4.96% of the population born in the USA are suffering from colorectal cancer [3]. Even in Asia, where the incidence rate is reported to be the lowest [4], the threat posed by colorectal cancer cannot be underestimated. Taking China as an example, the incidence and mortality of colorectal cancer there have kept rising. China’s cancer statistics manifest that the incidence and mortality of colorectal cancer ranked fifth among all malignant tumors in China, bringing about 380,000 new cases and 190,000 deaths annually. When they are seeking medical examination, most patients have already been in the advanced stage [5, 6]. Despite the continuous development of treatment technology, the 5-year survival rate of patients with metastatic disease is still less than 10% [7], which is probably due to the inability to diagnose early and the lack of specific markers to determine tumor development or patients’ prognosis. Therefore, to enhance the prognosis of patients with colorectal cancer, it is indispensable to explore effective diagnostic and therapeutic methods.

Programmed cell death protein 1 (PD-1), a sort of inhibitory checkpoint molecule, was discovered and named by Japanese scholar Ishida in 1992 [8]. It belongs to the CD28 family and is expressed on the surface of activated T cells to regulate proliferation and activation [9]. PD-L1 (also known as B7-H1) is the dominant ligand for PD-1 and expressed in activated T cells, B cells, dendritic cells, macrophages, endothelial cells, and a significant number of tumor cells [10]. In the healthy immune system, the activation of the PD-1/PD-L1 pathway can inhibit the immune function of T lymphocytes and promote the inhibitory function of regulatory T cells, which can reduce the immune response of the body to normal peripheral tissues. Consequently, it can inhibit autoimmune responses, prevent the development of autoimmune diseases, and maintain autoimmune tolerance in healthy individuals [11]. When cancer occurs, the tumor cells will reduce their immunogenicity by expressing PD-L1. Hence, they will not be recognized by the immune system and will evade immune attack [12]. In a variety of tumors, the PD-L1 expression is usually associated with poor prognosis [13, 14].

Current theories on the expression of PD-L1 in colorectal cancer and tumor prognosis are limited and controversial. Some studies have manifested the palpable connection between PD-L1 expression and overall survival rate of colorectal cancer patients [15–18], but the others utter the contradictory statement [19, 20]. So, we used meta-analysis to analyze the prognostic value of programmed death factor ligand 1 (PD-L1), which will also lay a theoretical foundation for the application of PD-1/PD-L1 immunoassay point inhibitors in colorectal cancer.

## Materials and methods

### Bibliographic search

Two authors independently searched PubMed, Web of Science, Embase, and Cochrane Library for published literature on PD-L1 and colorectal cancer. Publication time of the included articles ranges from the time when the database was established until August 2018. All publications are in English. Search strategies are (“colorectal neoplasms” OR “colorectal cancer” OR “colorectal carcinoma” OR “colorectal cancers” OR “colonic neoplasms” OR “rectal neoplasms”) AND (“PD-1” OR “PD-L1” OR “programmed death 1” OR “programmed death ligand 1” OR “programmed cell death ligand 1” OR “programmed death 1 ligand 1” OR “B7-H1” OR “CD274”).

### Inclusion criteria

The following are the inclusion criteria:

1. The clinical and pathological data of all cases are complete, and all were diagnosed as colorectal cancer by pathological examination;
2. Detecting the PD-L1 expression in colorectal cancer tissues by immunohistochemical staining;
3. The literature provides the relationship between PD-L1 expression and overall survival (OS) in patients with colorectal cancer;
4. The literature provides the relationship between PD-L1 expression and clinicopathological features, such as primary tumor size, clinical stage, and differentiation;
5. The literature provides sufficient information to estimate the hazard ratio (HR).

### Exclusion criteria

The following are the exclusion criteria:

1. The included literature is not an original study;
2. The data contained in the research is wrong, or the quality of the incorporated literature is low;
3. The included literature is based on animal or cell experiments;
4. Cannot use the data provided in the literature to calculate the hazard ratio (HR) associated with PD-L1;
5. The included literature did not analyze the expression of PD-L1 in tumor cells.

### Data extraction and quality evaluation

Two authors independently screened and extracted data found on inclusion and exclusion criteria and discussed together or adjudicated by third parties in case of disagreement. For the lack of information, we contacted the original author as much as possible. Extracted contents included author, publication year, country, positive threshold, follow-up period, baseline and clinicopathological information of patients, hazard ratio (HR), and 95% confidence interval (95%CI) related to PD-L1 expression.

Methodological quality assessment of the included data was carried out using the Newcastle-Ottawa Scale (NOS). Scores of the NOS are split into three aspects: object selection, inter-group comparability, and outcome measurement. The highest rating is 9 points, and the study with more than 6 points is considered as a high-quality one [21].

### Statistical analysis

We implemented the meta-analysis based on the Preferred Reporting Items for Systematic Review and Meta-Analysis (PRISMA) Checklist (Additional file 1: Table S1). The hazard ratio (HR) and 95%CI of each study were combined to assess the relationship between the expression of PD-L1 and the prognosis, but the correlation between the PD-L1 expression and clinicopathological features was calculated using the pooled odds ratio (OR) and 95%CI. The heterogeneity test was performed using the *χ*^2^ test, and then we utilized the fixed-effect model or the random-effect model according to heterogeneity. All the analyses above were presented by Revman 5.3 software.

All the studies are retrospective cohort studies, whose heterogeneity is often inevitable. Therefore, based on the analyses above, we carried out publication bias test and sensitivity analysis with the Stata 12.0 software to explore the sources of heterogeneity. And according to Begg’s or Egger’s test, *P* > 0.05 manifested that there was no publication bias in the study.

## Results

### Data collection and characteristics

**Fig. 1:**
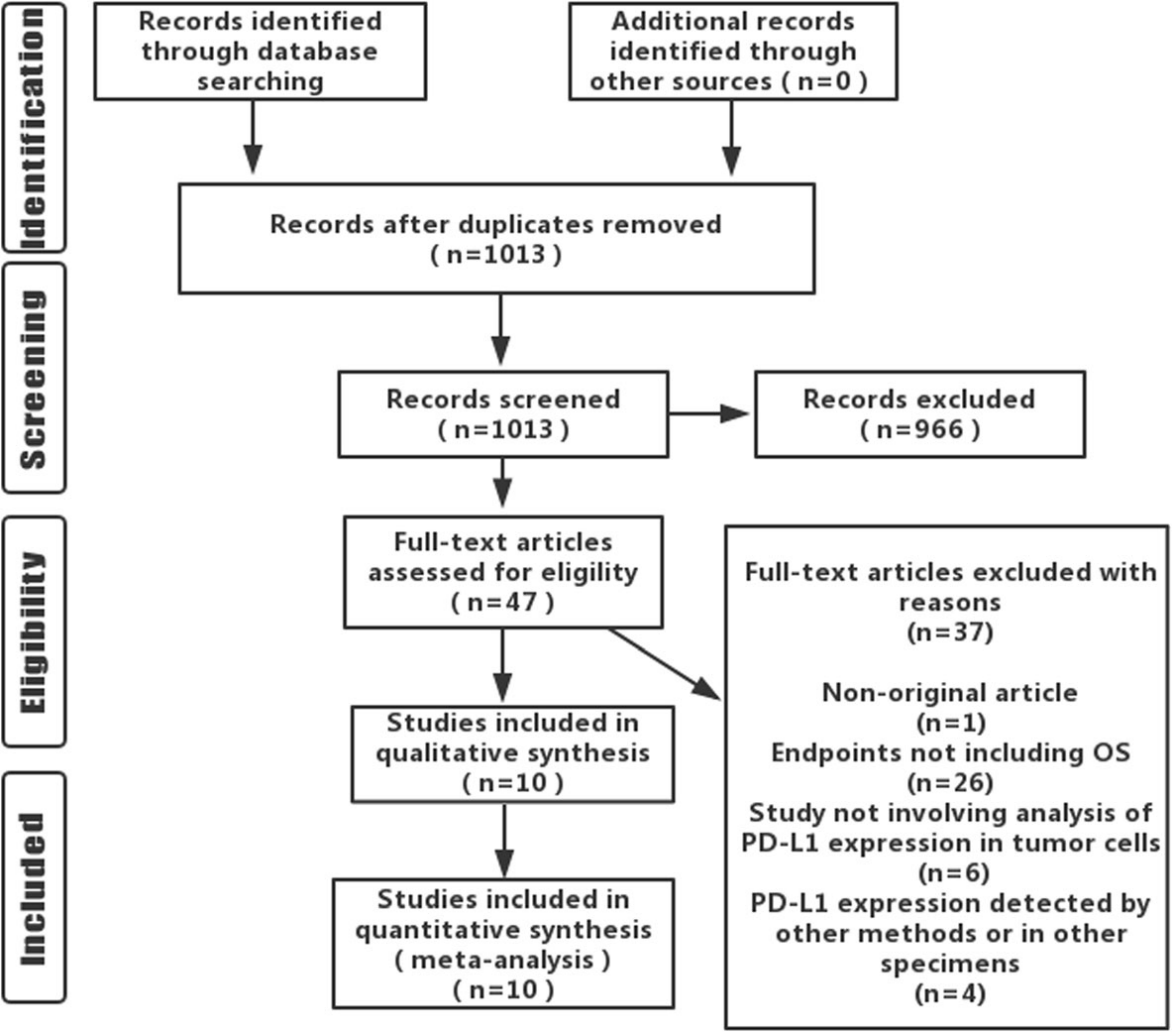
Flowchart of literature screening

A total of 1013 related articles were initially retrieved. After the layer-by-layer screening, 10 items were ultimately included, totaling 3481 cases (Fig. 1). The basic characteristics of the included studies were presented in Table 1. The NOS was used to estimate the quality of the included studies, and all were proved to be high-quality ones (Table 2).

**Table 1:** Basic characteristics of included studies

Author, year	Country	No.	Stage	Follow-up	Preoperative chemoradiotherapy	Curative surgical resection	Postoperative adjuvant chemotherapy no.	PD-L1 (%)	HR	Cutoff for positive
Enkhbat, 2018 [15]	Japan	116	II–III	52 months (mean)	NA	YES	57	52/116	3.873	Score > 3 (intensity + area)
Masugi, 2016 [35]	America	450	I–IV	> 5 years	NA	YSE	NA	121/450	1.124	Score = 1 (intensity)
								117/450	0.980	Score = 2 (intensity)
								139/450	1.042	Score = 3 (intensity)
								26/450	1.370	Score = 4 (intensity)
Saigusa, 2016 [16]	Japan	90	I–IV	> 6 months	YES	YES	90	36/90	2.452	Score ≥ 2 (intensity)
Zhu, 2015 [36]	China	120	NA	39 months (mean)	NA	YES	NA	28/120	0.692	Score > 4 (intensity + area)
Liang, 2014 [17]	China	185	I–IV	> 5 years	NA	YES	NA	102/185	1.740	Score > 4 (intensity + area)
Droeser, 2014 [37]	Switzerland	1420	NA	> 5 years	NA	YES	NA	495/1420	0.92	Subjective evaluation
Hamada, 2017 [20]	America	384	I–IV	> 5 years	NA	YES	NA	211/384	1.20	Score ≥ 2 (intensity + area)
Lee, 2018 [18]	Korea	336	I–IV	52 months (mean)	NA	YES	NOT	15/336	3.785	Area > 1%
Li, 2016 [19]	China	276	NA	61 months (mean)	NA	YES	189	138/276	1.048	Score > 4 (intensity + area)
Miller, 2017 [38]	Australia	104	III	82.5 months (mean)	NA	YES	89	60/104	1.00	Subjective evaluation

*NA* not available, *NOT* no patients underwent, *mean* average follow-up time is provided only, *intensity + area* the score involved staining intensity and staining range, *intensity* the score involved staining intensity only

**Table 2:** Newcastle-Ottawa Scale for quality assessment

Author, year	Selection				Comparability	Outcome			Total score
	Exposed cohort	Non-exposed cohort	Ascertainment of exposure	Outcome of interest	Control for factor	Assessment of outcome	Follow-up long enough	Adequacy of follow-up	
Enkhbat, 2018 [15]	*	*	*		**		*	*	7
Masugi, 2016 [35]	*	*	*		**		*	*	7
Saigusa, 2016 [16]	*	*	*		**		*	*	7
Zhu, 2015 [36]	*	*	*		**		*	*	7
Liang, 2014 [17]	*	*	*	*	**		*	*	8
Droeser, 2014 [37]	*	*	*		**		*	*	7
Hamada, 2017 [20]	*	*	*		**		*	*	7
Lee, 2018 [18]	*	*	*		**		*	*	7
Li, 2016 [19]	*	*	*		**		*	*	7
Miller, 2017 [38]	*	*	*		**		*	*	7

*The article scored one point in the project

**The article scored two points in the project

### Relationship between PD-L1 expression and prognosis of colorectal cancer

**Fig. 2:**
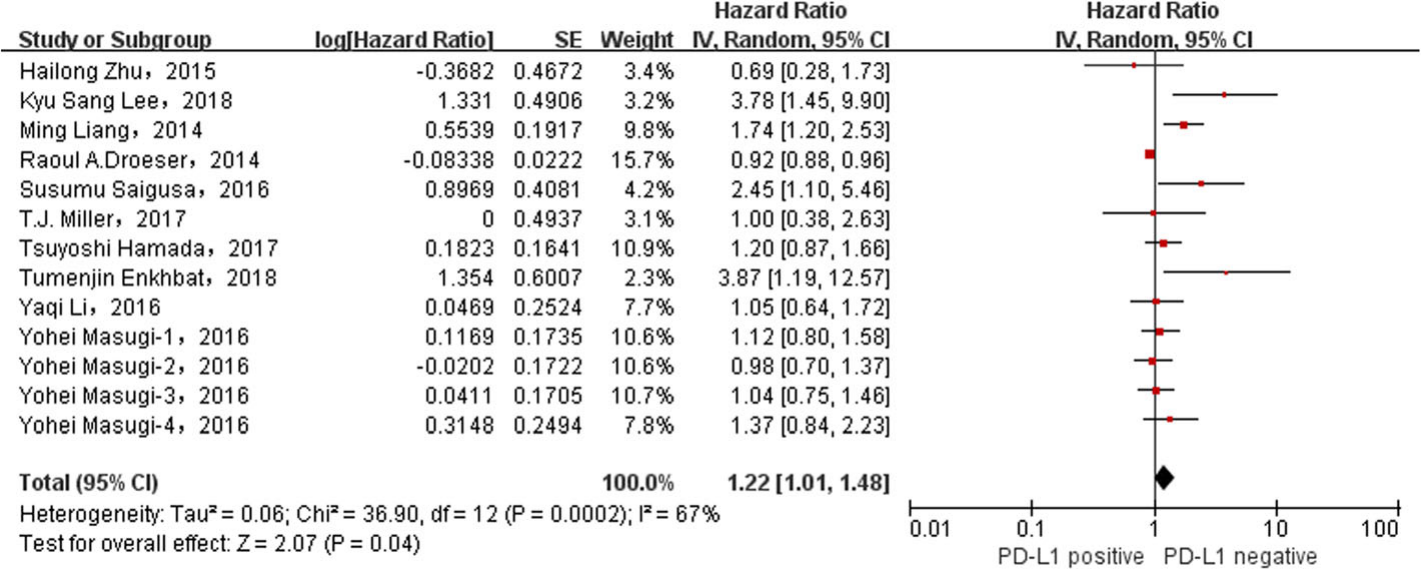
Forest plot describing the relationship between PD-L1 expression and prognosis in colorectal cancer

The relationship between the overexpression of PD-L1 and the poor prognosis of colorectal cancer patients was evaluated, and the consequence displayed a significant correlation (HR = 1.22, 95%CI = 1.01–1.48, *P* = 0.04, random effect) (Fig. 2).

### Relationship between PD-L1 expression and clinicopathological features of colorectal cancer

Apropos of clinicopathological features, the merged odds ratio (OR) exhibited that highly expressed PD-L1 was firmly related to lymphatic invasion (OR = 3.49, 95%CI = 1.54–7.90, *P* = 0.003) and advanced stage (OR = 1.77, 95%CI = 1.41–2.23, *P* < 0.00001), but not correlative with patients’ gender, microsatellite instability, or tumor location (Table 3) (Additional file 2: Figure S1).

**Table 3:** The relationship between PD-L1 expression and clinicopathological characteristics in subgroup analysis

Subgroup analysis	No. of studies	No. of patients	Experimental group: positive/total	Control group: positive/total	OR	95%CI	*P* value	Heterogeneity (*I*^2^), %	Begg’s test (*P* value)	Egger’s test (*P* value)
Gender	9	3706	Male	Female	0.92	0.79–1.07	0.29	0	0.917	0.639
			898/1852	1018/1854						
The situation of primary tumor	7	2809	T3+T4	T1+T2	1.15	0.68–1.95	0.60	70	0.764	0.115
			1018/2176	347/633						
The involvement of regional lymph nodes	6	2652	N1+N2	N0	1.32	0.84–2.09	0.23	74	0.060	0.012
			558/1225	729/1427						
Stage	6	2064	III+IV	I+II	1.77	1.41–2.23	P < 0.00001	23	0.452	0.512
			594/936	659/1128						
Vascular invasion	6	2052	Positive	Negative	1.12	0.72–1.75	0.62	63	0.133	0.090
			201/564	491/1488						
Tumor location	6	3133	Left	Right	0.86	0.53–1.40	0.55	82	0.452	0.283
			916/1873	711/1260						
Microsatellite instability	4	2012	MSI-H	MSI-L+MSS	0.95	0.46–1.97	0.89	84	1.000	0.347
			223/362	1012/1650						
Lymphatic invasion	4	723	Positive	Negative	3.49	1.54–7.90	0.003	73	1.000	0.764
			122/280	83/443						
Tumor differentiation	4	1862	Poor	Well to moderate	1.90	0.55–6.63	0.31	88	0.734	0.292
			108/156	1057/1706						
Mucinous properties	3	1563	Mucinous	Other	0.94	0.42–2.10	0.88	56	–	–
			34/108	573/1455						
Grade	3	1562	III	I+II	0.66	0.42–1.03	0.07	52	–	–
			86/239	569/1323						

### Subgroup analysis of heterogeneity sources

As for subgroup analysis of heterogeneity sources, the heterogeneity of each subgroup decreased in varying degrees (Additional file 3: Figure S2). Among them, the non-Asian group had the minimum heterogeneity (*I*^2^ = 12% < 50%). In addition, the results of the Asian group (HR = 1.73, 95%CI = 1.10–2.73, *P* = 0.02, *I*^2^ = 60%), the non-Asian group (HR = 0.93, 95%CI = 0.89–0.97, *P* = 0.001, *I*^2^ = 12%), and the tumor stages I–IV group (HR = 1.32, 95%CI = 1.06–1.63, *P* = 0.01, *I*^2^ = 52%) were still statistically significant, but other subgroup analyses failed to arrive at such a statistically significant conclusion (Table 4).

**Table 4:** Subgroup analysis of heterogeneity sources

		No. of studies	HR	95%CI	*P* value	Heterogeneity (*I*^2^), %
Country	Asian	6	1.73	1.10–2.73	0.02	60
	Non-Asian	4	0.93	0.89–0.97	0.001	12
Stages	I–IV	5	1.32	1.06–1.63	0.01	52
Follow-up period	≥ 5 years	4	1.12	0.94–1.34	0.20	65
	< 5 years	6	1.62	0.93–2.82	0.09	61
Postoperative adjuvant chemotherapy		4	1.61	0.88–2.94	0.12	54
Sample size	≥ 200	5	1.09	0.92–1.28	0.32	54
	< 200	5	1.61	0.99–2.60	0.05	47

### Publication bias analysis and sensitivity analysis

**Fig. 3:**
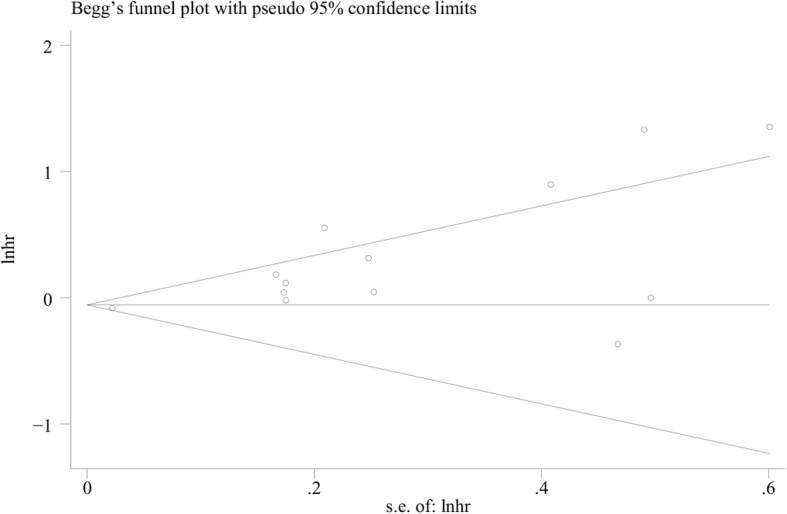
Begg’s funnel plot for publication bias test including PD-L1 expression and prognosis in colorectal cancer

**Fig. 4:**
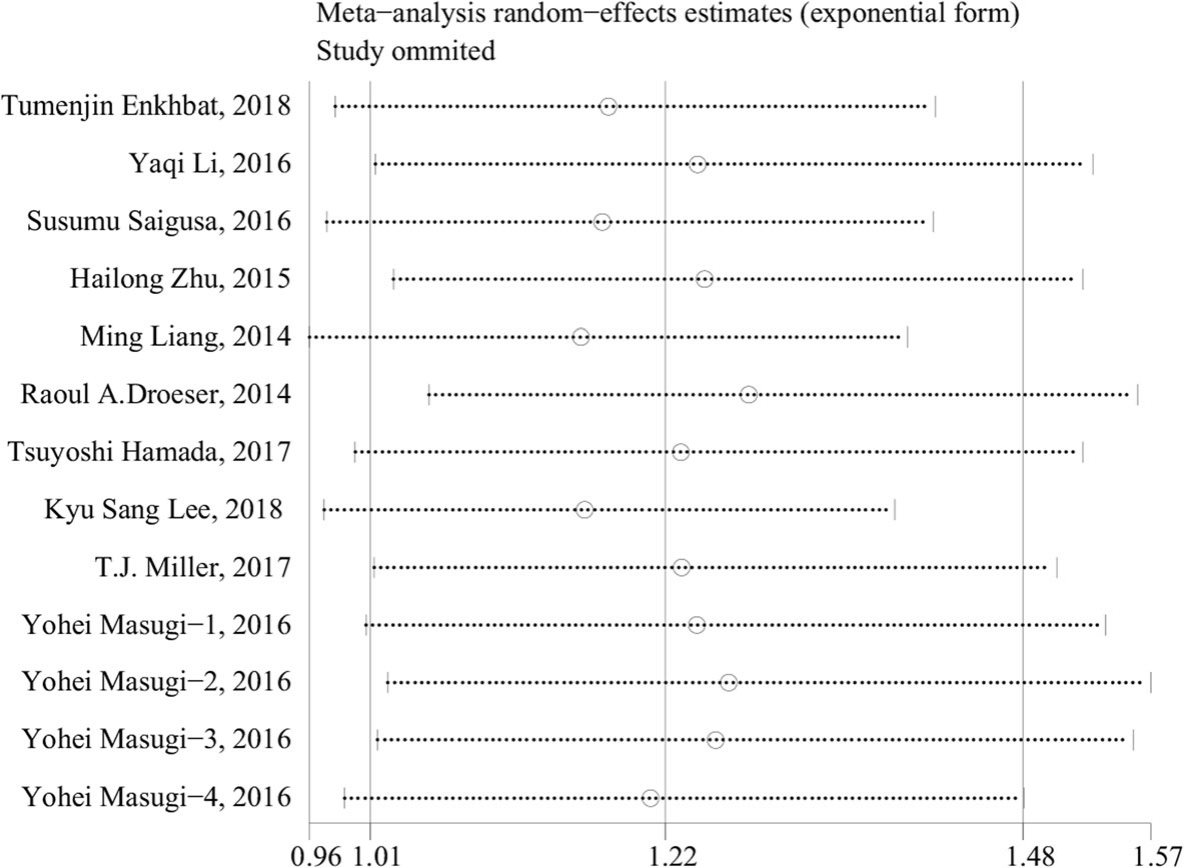
Sensitivity analysis including PD-L1 expression and prognosis in colorectal cancer

The funnel plot is a conventional method to evaluate whether there is a “publication bias” in the meta-analysis, but as a qualitative judgment, its subjectivity makes different observers come to different conclusions [22]. Given this, Begg’s test [23] and Egger’s test [24] were created to evaluate “publication bias” quantificationally. In this meta-analysis, according to Begg’s test (*P* = 0.428 > 0.05), there was no publication bias in the included literature involving PD-L1 and OS (Fig. 3). The detection results of publication bias in subgroup analyses are shown in Table 2 and Additional file 4: Figure S3. Sensitivity analysis pointed out that the conclusions were generally stable (Fig. 4 and Additional file 5: Figure S4).

## Discussion

PD-1 and PD-L1 are inhibitory costimulatory molecules. When tumor cells express PD-L1 to combine with the PD-1 provided by tumor-infiltrating lymphocytes, the immune effect of T cells in the tumorous microenvironment is inhibited, which mediates the occurrence of tumor immune escape and promotes the progress of cancer [25]. At present, a lot of research has been done on this pair of inhibitory costimulatory molecules, but the regulatory mechanism of the signaling pathway in colorectal carcinoma has not been clarified, and theories in many fields are controversial. Although some systematic reviews focused on the prognostic value of PD-L1 in all types of solid tumors also mentioned the relationship between PD-L1 and prognosis of colorectal cancer in passing [26–28], yet their results in this regard had limitations because of the lack of in-depth research. Wu et al. suggested that PD-L1 overexpression was positively correlated with 5-year OS deterioration in colorectal cancer, but they only included two papers and used OR value to evaluate the results, meaning the existence of great bias [28]. Different from the above conclusion, Pyo et al. applied HR involving PD-L1 to assess the relevance between PD-L1 expression and prognosis of colorectal cancer and then concluded that there was no connection between them. However, they merely contained four retrospective studies, which lacked persuasiveness [27]. Xiang et al. also argued that PD-L1 could not be used as a prognostic indicator of colorectal cancer, but they misapplied risk ratio (RR), a specific measure for evaluating prospective studies, to the analysis of retrospective studies, so the findings should be treated cautiously [26]. Therefore, to resolve the controversy and deficiency mentioned above, this meta-analysis comprehensively collected relevant literature based on the inclusion criteria and adopted the hazard ratio (HR) associated with PD-L1 to estimate the prognostic value of PD-L1 in colorectal cancer. In addition to that, we also explored the relationship between the expression of PD-L1 and the clinicopathological characteristics of colorectal cancer to make the outcome more convincing.

This meta-analysis demonstrated that PD-L1 expression could be utilized as an independent factor in judging the prognosis of colorectal cancer (HR = 1.22, 95%CI = 1.01–1.48, *P* = 0.04, random effect). Nevertheless, there was inevitable heterogeneity among the retrospective studies included in this meta-analysis (*P* = 0.0002, *I*^2^ = 67%). In order to make the conclusion more persuasive and scientific, we adopted the subgroup analysis to explore the heterogeneity sources. And as the findings suggested, the heterogeneity of each subgroup decreased in varying degrees, indicating that these factors have certain degrees of influence. Among them, the non-Asian group had the minimum heterogeneity (*I*^2^ = 12% < 50%), which implied the essential role the regional or ethnic differences played on engendering heterogeneity.

As the subgroup analysis of PD-L1 and clinicopathological features indicated, PD-L1 overexpression in colorectal cancer cells was associated with lymphatic invasion. Previous experimental studies have shown that they are indeed relevant. Epithelial-to-mesenchymal transition (EMT) leads to lymphatic invasion [29], and the expression of PD-L1 in tumor cells facilitates immunosuppression, both of which contribute to tumor progression and metastasis. MiR-200/Zinc finger E-box-binding homeobox 1 (ZEB1) was initially known as EMT regulatory axis, and the bidirectional negative feedback regulation mechanism between ZEB1 and miR-200 makes the corresponding cells actualize EMT [30]. But recently, the mechanism that miR-200/ZEB1 axis can also regulate PD-L1 to facilitate immunosuppression has been proved by experiments [31]. Therefore, the relationship between lymphatic invasion and PD-L1 overexpression can be considered to be mutually “parallel.” Also, consistent with another inference of this meta-analysis that the inhibition of the PD-1/PD-L1 signaling pathway in advanced colorectal cancer could achieve remarkable results, existing clinical trials have exhibited the high security and activity of the treatment with PD-1/PD-L1 immunocheckpoint inhibitors [32].

## Limitations

To mention first, all the included articles were retrospective studies, whose bias could not be eliminated, so the consequences were generally stable. Also, it should be noted that all the articles included are in English, meaning the lack of research especially those negative studies in non-English speaking countries, which leads to the absence of representativeness and the production of bias.

Secondly, it has been reported that the expression of PD-L1 in tumor cells is a critical factor in making the monoclonal antibody against PD-1/PD-L1 effective. However, in every article, the threshold of PD-L1 positive is different and brings about a tremendous impact on the experimental results. According to published articles, patients with PD-L1 positive can obtain a better outcome in the treatment with immunocheckpoint inhibitors as the threshold increases [33]. So, uniformly applying the most suitable PD-L1 positive threshold to the following research should be a top priority. Besides that, using of different immunohistochemical antibodies in various studies leads to specific errors.

Furthermore, the latest experiments indicate that cancer patients [34] with intestinal flora disorders have a worse prognosis in the treatment with anti-PD-1/PD-L1 antibodies, which can be concluded that intestinal flora balance plays a vital role in the efficacy of PD-1/PD-L1 immunocheckpoint inhibitors. Therefore, the following study in this field should take intestinal flora into consideration.

## Conclusions

This study analyzed all the available interrelated information in the published literature and exhibited that the expression of PD-L1 was significantly correlated with the overall survival rate of colorectal cancer. The more the PD-L1 was expressed, the worse prognosis the colorectal cancer patients would undergo. Concerning clinicopathological features, the expression of PD-L1 was bound up with lymphatic invasion and tumor stage, but not gender, microsatellite instability, or tumor differentiation. In other words, the expression of PD-L1 could be utilized as an independent factor in judging the prognosis of colorectal cancer, and patients with advanced cancer or lymphatic invasion were more likely to express PD-L1. This conclusion may lay a theoretical foundation for the application of PD-1/PD-L1 immunoassay point inhibitors but still need to be verified by sizeable well-designed cohort studies.

## Additional files

Additional file 1: **Table S1.** PRISMA 2009 Checklist. (DOC 53 kb)

Additional file 2: **Figure S1.** Forest plots assessing the relationship between PD-L1 and clinicopathological characteristics: (a) gender; (b) grade; (c) lymphatic invasion; (d) microsatellite instability; (e) mucinous properties; (f) stage; (g) the involvement of regional lymph nodes; (h) tumor differentiation; (i) tumor location; (j) the situation of primary tumor; (k) vascular invasion. (PNG 220 kb)

Additional file 3: **Figure S2.** Subgroup analysis of heterogeneity sources: (a) Asian; (b) non-Asian (c) stages I–IV; (d) follow-up more than 5 years; (e) follow-up less than 5 years (f) postoperative adjuvant chemotherapy; (g) sample size ≥ 200; (h) sample size < 200. (PNG 287 kb)

Additional file 4: **Figure S3.** Detection of publication bias in subgroup analysis: (a) gender; (b )lymphatic invasion; (c) microsatellite instability; (d) stage; (e) the involvement of regional lymph nodes; (f) the situation of primary tumor; (g) tumor location; (h) tumor differentiation; (i) vascular invasion. (PNG 99 kb)

Additional file 5: **Figure S4.** Sensitivity analysis of subgroup analysis: (a) gender; (b) lymphatic invasion; (c) microsatellite instability; (d) stage; (e) the involvement of regional lymph nodes; (f) the situation of primary tumor; (g) tumor location; (h) tumor differentiation; (i) vascular invasion. (PNG 147 kb)

## Abbreviations

CI: Confidence interval
EMT: Epithelial-to-mesenchymal transition
HR: Hazard ratio
NOS: Newcastle-Ottawa Scale
OR: Odds ratio
OS: Overall survival
PD-1: Programmed cell death protein 1
PD-L1: Programmed death factor ligand 1
PRISMA: Preferred Reporting Items for Systematic Review and Meta-Analysis
ZEB1: Zinc finger E-box-binding homeobox 1
