# Supplementary figures and images for: Clinicopathological and prognostic significance of PD-L1 expression in colorectal cancer: a systematic review and meta-analysis
Source: World J Surg Oncol. 2019 Jan 4;17:4. doi: 10.1186/s12957-018-1544-x (PMC6320581; doi:10.1186/s12957-018-1544-x)

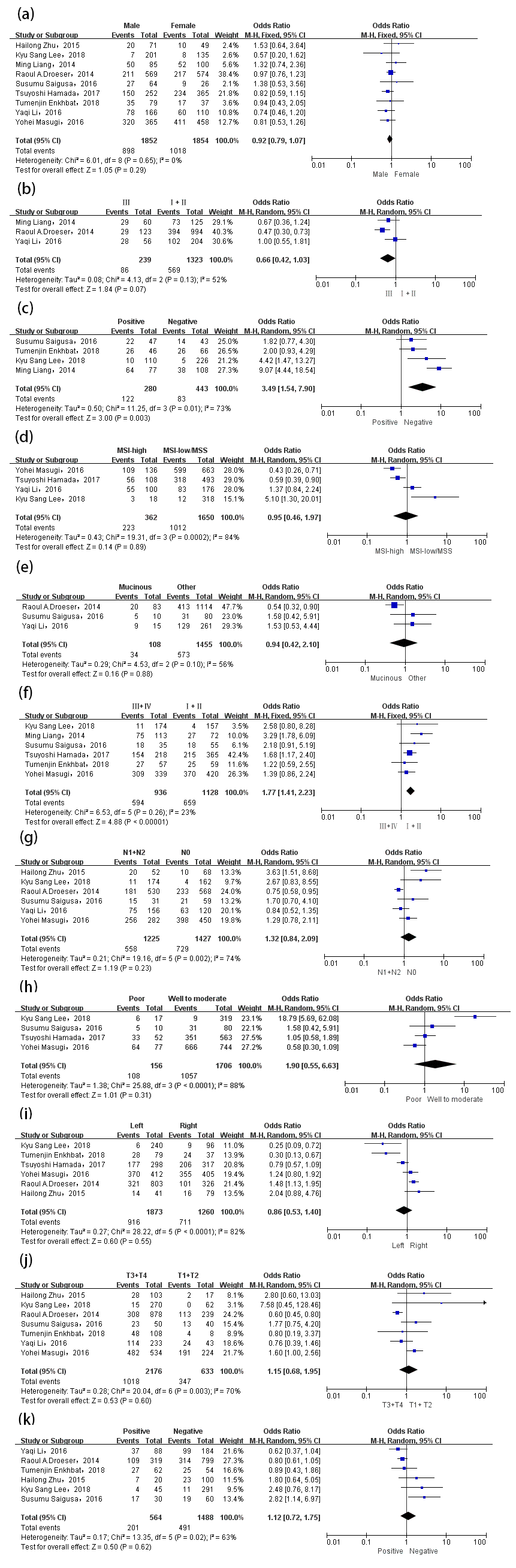

Supplement: Supplementary file 2 — Figure S1. Forest plots assessing the relationship between PD-L1 and clinicopathological characteristics: (a) gender; (b) grade; (c) lymphatic invasion; (d) microsatellite instability; (e) mucinous properties; (f) stage; (g) the involvement of regional lymph nodes; (h) tumor differentiation; (i) tumor location; (j) the situation of primary tumor; (k) vascular invasion. (PNG 220 kb) [file 12957_2018_1544_MOESM2_ESM.png]

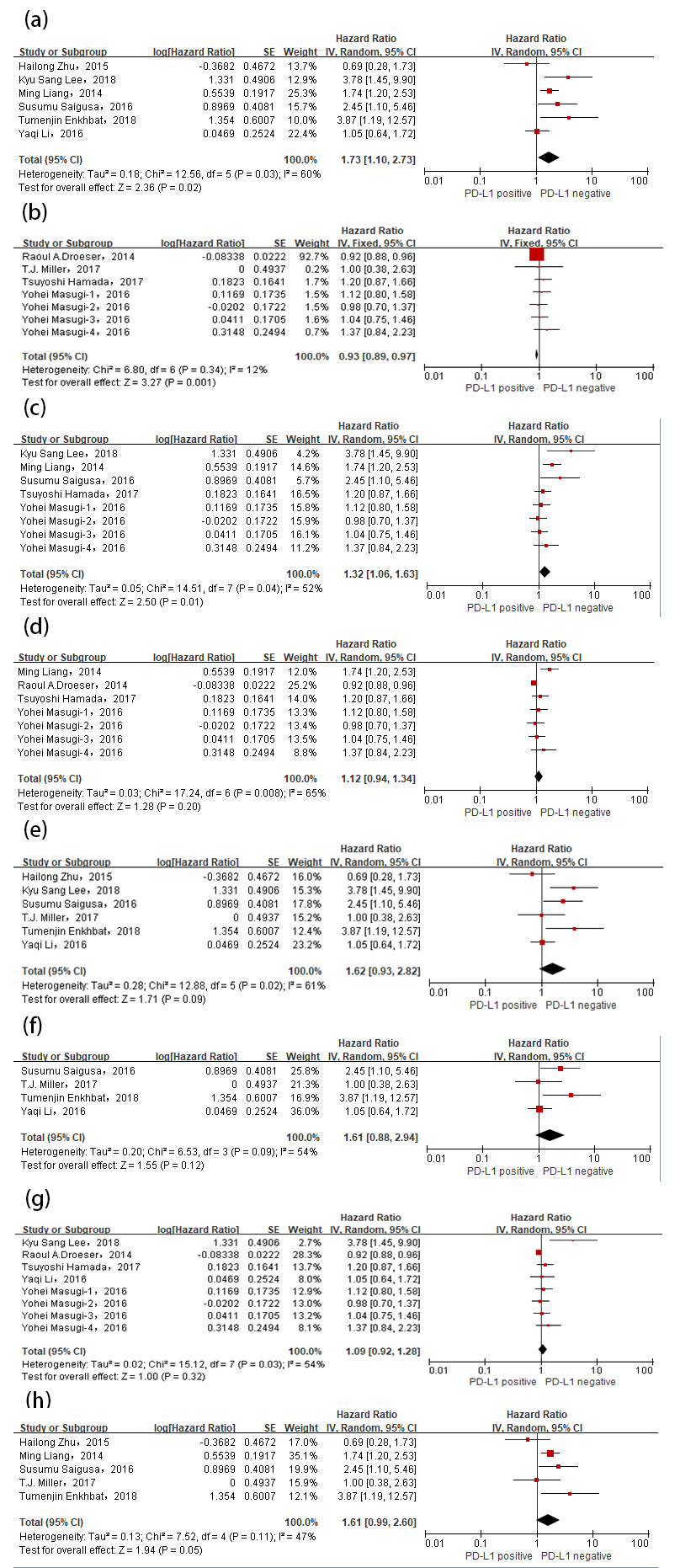

Supplement: Supplementary file 3 — Figure S2. Subgroup analysis of heterogeneity sources: (a) Asian; (b) non-Asian (c) stages I–IV; (d) follow-up more than 5 years; (e) follow-up less than 5 years (f) postoperative adjuvant chemotherapy; (g) sample size ≥ 200; (h) sample size < 200. (PNG 287 kb) [file 12957_2018_1544_MOESM3_ESM.png]

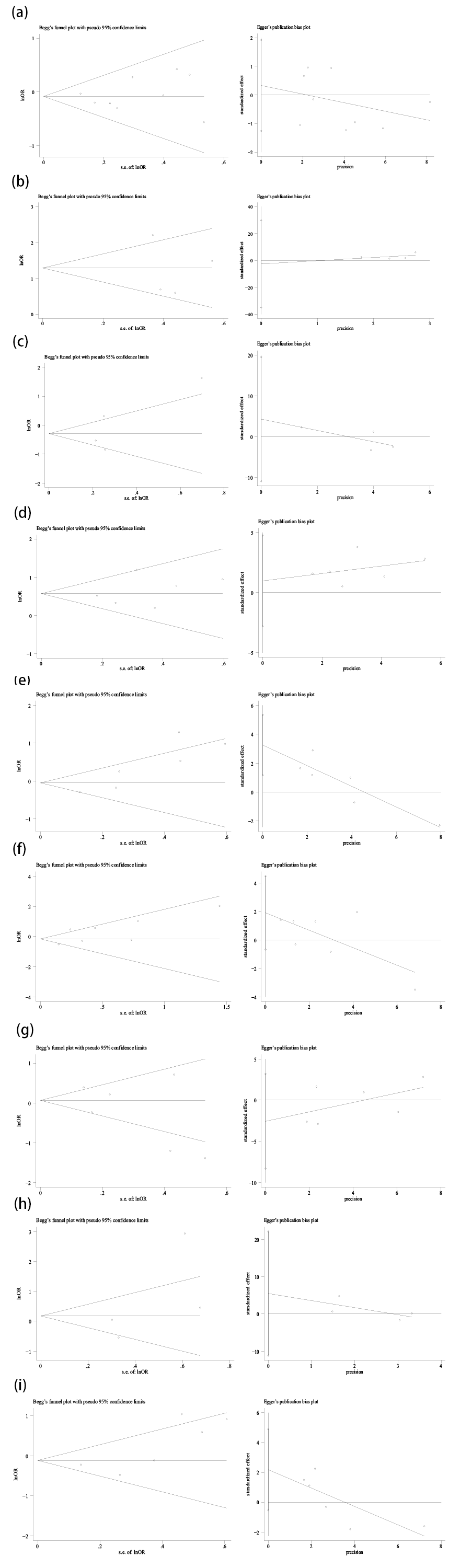

Supplement: Supplementary file 4 — Figure S3. Detection of publication bias in subgroup analysis: (a) gender; (b )lymphatic invasion; (c) microsatellite instability; (d) stage; (e) the involvement of regional lymph nodes; (f) the situation of primary tumor; (g) tumor location; (h) tumor differentiation; (i) vascular invasion. (PNG 99 kb) [file 12957_2018_1544_MOESM4_ESM.png]

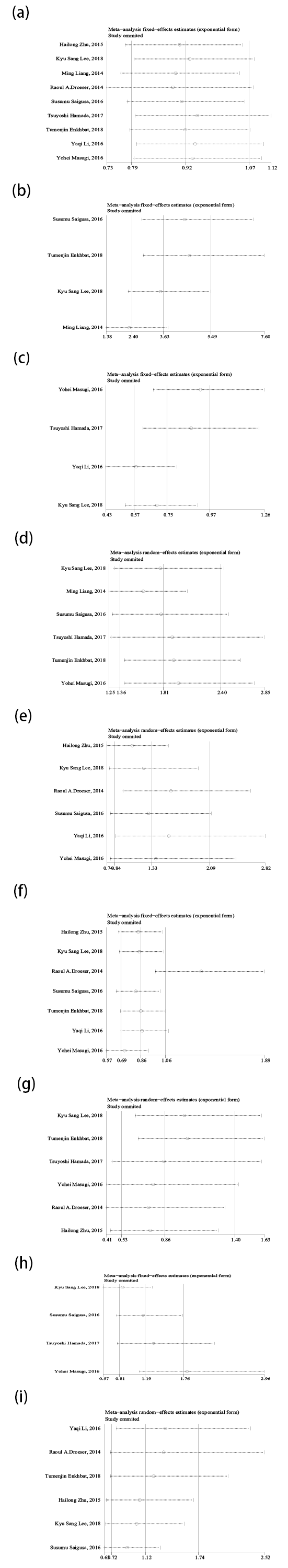

Supplement: Supplementary file 5 — Figure S4. Sensitivity analysis of subgroup analysis: (a) gender; (b) lymphatic invasion; (c) microsatellite instability; (d) stage; (e) the involvement of regional lymph nodes; (f) the situation of primary tumor; (g) tumor location; (h) tumor differentiation; (i) vascular invasion. (PNG 147 kb) [file 12957_2018_1544_MOESM5_ESM.png]
